# A multi‐faceted intervention to reduce alcohol misuse and harm amongst sports people in Ireland: A controlled trial

**DOI:** 10.1111/dar.12585

**Published:** 2017-08-07

**Authors:** Anne O'Farrell, Melanie Kingsland, Susan Kenny, Nazih Eldin, John Wiggers, Luke Wolfenden, Shane Allwright

**Affiliations:** ^1^ Health Intelligence Unit, Health and Wellbeing Directorate, Health Services Executive, Stewarts Hospital Dublin Ireland; ^2^ School of Medicine and Public Health The University of Newcastle Newcastle Australia; ^3^ Hunter Medical Research Institute Newcastle Australia; ^4^ National Office for Suicide Prevention Health Services Executive, Stewarts Hospital Dublin Ireland; ^5^ Health Promotion Department Dublin & North East, Navan Co. Meath Ireland; ^6^ Hunter New England Population Health Newcastle Australia; ^7^ Department of Public Health and Primary Care, School of Medicine, Trinity College Dublin the University of Dublin Dublin Ireland

**Keywords:** alcohol drinking, sport, intervention, controlled trial

## Abstract

**Introduction and Aims:**

Alcohol misuse and harm are more prevalent amongst sports people than non‐sports people. Few studies have trialled interventions to address alcohol misuse for this group. The study aimed to test the effectiveness of an intervention to reduce alcohol misuse and related harms amongst amateur sports people in Ireland.

**Design and Methods:**

A controlled trial was conducted in two counties in Ireland. A random selection of sports clubs in one county received a 4 month multi‐faceted intervention. All sports clubs in a non‐adjacent county acted as control sites. Consumption of more than 21 units of alcohol per week and six or more standard drinks on a single occasion at least once per week was the primary study outcome. Alcohol Use Disorders Identification Test scores and number of alcohol‐related harms were also reported. Outcomes were assessed for cross‐sectional samples of players at pre‐intervention and post‐intervention and paired samples of players who completed surveys at both times. Generalised linear mixed model analysis was used.

**Results:**

There was no evidence of effect for the primary outcomes or Alcohol Use Disorders Identification Test scores. There was a statistically significant difference in the median number of alcohol‐related harms reported by intervention group players compared with control group players at post‐intervention for the paired samples [intervention: 0; control: 3; incident rate ratio 0.56 (0.37, 0.84); P = 0.005].

**Discussion and Conclusions:**

Intervention in community sports clubs may be effective in reducing the number of alcohol‐related harms. Low levels of intervention participation and inadequate intervention dose are possible reasons for lack of a broader intervention effect. [O'Farrell A, Kingsland M, Kenny S, Eldin N, Wiggers J, Wolfenden L, Allwright S. A multi‐faceted intervention to reduce alcohol misuse and harm amongst sports people in Ireland: A controlled trial. *Drug Alcohol Rev* 2018;37:14–22]

## Introduction

Ireland has a history of excessive alcohol consumption, with average annual consumption peaking in 2001 at 14.4 l of pure alcohol per adult aged 15 years and over 1. Although average annual consumption had fallen and stabilised at around 11 l in 2013, this was almost 20% more than the Organisation for Economic Co‐operation and Development average of 8.9 l 2. The prevalence of heavy episodic drinking in Ireland is also high 3. In 2014–2015, 57% of males and 21% of females who consumed alcohol reported drinking six or more drinks on a typical drinking occasion, 41% indicated that they did so at least once a month, and 24% reported doing so at least once a week 4.

The link between alcohol misuse, risk of alcohol‐related harms and sport is well established, with people who participate in sport being more likely to engage in alcohol misuse than non‐sports people 5, 6. High levels of alcohol misuse have been found amongst amateur and professional sports people in several countries, including New Zealand 6, Australia 7, the USA 8 and Brazil 9. People involved in team sports have been suggested to be at greater risk of alcohol misuse and alcohol‐related harm than those involved in non‐team sports 7, 10, 11, 12.

In Ireland, amateur Gaelic football and hurling players report high levels of alcohol consumption, heavy episodic drinking and alcohol‐related harms. For instance, 54% of players have reported regular heavy episodic drinkers (six or more drinks at least once a week) compared with 40% of males nationally 13. Thirty percent of such players also reported drinking more than the recommended weekly level of alcohol (21 units) compared with 15% nationally, while 32% reported involvement in a violent incident due to their drinking, compared with 15% nationally 13.

Community‐level interventions have been shown to be effective in reducing alcohol‐related harm including drinking and driving, alcohol‐related traffic fatalities and assault injuries 14, 15, 16, 17. Successful interventions have employed a multi‐faceted approach to the prevention of alcohol‐related harms and have focused on the community as a system involving the individual drinker, groups of drinkers and drinking environments 17.

Although the sport setting has potential for inclusion in such community‐level interventions, a Cochrane systematic review on interventions implemented through sporting organisations to improve alcohol misuse and harms found no controlled trials 18. Subsequent to this review, only one randomised controlled trial of an alcohol‐harm reduction intervention in community sports clubs has been reported 19. The trial was conducted in amateur football clubs in Australia and involved a 2.5 year multi‐faceted, socio‐ecological intervention to reduce risky alcohol consumption and alcohol‐related harms through changing club alcohol management practices. The intervention was effective in reducing risky alcohol consumption [5+ standard drinks (10 g/alcohol) per occasion at least once a month] by club members while at the club [intervention: 19%; control: 24%; odds ratio 0.63 (95% confidence interval 0.40 to 1.00); *P* = 0.05] and risk of alcohol‐related harm overall [Alcohol Use Disorders Identification Test (AUDIT) score ≥8; intervention: 38%; control: 45%; odds ratio 0.58 (95% confidence interval 0.38 to 0.87); *P* < 0.01] 19. Given significant differences between countries in the cultural, social and structural characteristics of sport, further research is required to establish whether such an intervention is effective in other countries and for other sporting codes and could be rolled out by policy makers and sporting bodies more broadly.

This study aimed to assess the effectiveness of a multi‐faceted intervention in reducing alcohol consumption and risk of alcohol‐related harms amongst Gaelic Athletic Association (GAA) club players.

## Methods

### Ethical approval

Ethical approval was obtained from the Research Ethics Committee of the Faculty of Public Health Medicine of Ireland and the Royal College of Physicians in Ireland.

### Study design and setting

A cluster controlled trial was undertaken involving GAA clubs in two counties (one intervention county, one control county) in the Republic of Ireland. To avoid contamination, two counties with non‐contiguous boundaries from two different provinces in Ireland were selected. Of these, the county nearer to the study team was selected for intervention for pragmatic reasons. The GAA is the largest amateur sporting and community organisation in Ireland (>2000 clubs/800 000 members) with clubs competing in Gaelic football and/or hurling leagues and championships from January to September 20. Clubs consist of junior, intermediate and senior (including intercounty) players.

### Participant eligibility and recruitment

#### Gaelic Athletic Association clubs

All GAA clubs (*n* = 29) in the control county and a random sample of 14 (20%) of the 70 GAA clubs in the intervention county were invited to participate. Random sampling of intervention clubs was undertaken as resource constraints meant that only 14 clubs could receive the intervention. However, to increase study power, all clubs within the control county were invited to participate. The random sequence for selecting intervention clubs was generated using a random number generator in Microsoft Excel. Consent for clubs to participate in the study was obtained from GAA County Boards.

#### Gaelic Athletic Association players

All male club players aged 16 years and over who were currently playing with the club were eligible to participate, and injured players were excluded. A list of eligible players was provided by each club.

### Intervention

The intervention was implemented over a 4 month period and consisted of: (i) alcohol education for players; (ii) alcohol education for coaches; (iii) alcohol policy training for club managers and coaches; and (iv) an awareness campaign. The intervention was based on successful, multi‐faceted community‐based interventions for the reduction of alcohol‐related harm 14, 15, 16, 17 and included educational and environmental strategies targeting players, club management and the wider club community. Refer to Table S1 for further detail. The intervention was delivered by trained health promotion personnel from the local health service provider (March to June 2008).

### Control group

Players from both control and intervention group clubs received an education session on sports nutrition that did not include any alcohol‐related content.

### Data collection procedures and measures

A self‐administered pen and paper questionnaire was used to collect baseline data (April 2006 to February 2008) and post‐intervention data (May and October 2008). The survey items were developed based on established and previously used measures of alcohol consumption 21, 22, 23 and pilot tested with players from a GAA club outside the study counties. Research personnel distributed surveys to players after one or two training sessions at each club. A questionnaire was also administered to the GAA manager on duty at the club on the night of the player survey to collect information on club characteristics and intervention fidelity.

#### Primary outcome measures—alcohol consumption

Primary study outcomes were the proportions of players consuming more than 21 units of alcohol per week and six or more standard drinks on a single occasion at least once per week. A modified version of the Quantity–Frequency Scale 21 was used to measure alcohol consumption in litres of pure alcohol. Further information is provided in Box S1. Grams of pure alcohol consumed were calculated assuming 4.5, 12.5 and 33% alcohol/volume for beer, wine and spirits respectively. A standard drink was defined as 12 g of alcohol.

#### Secondary outcome measures—alcohol‐related harm

Alcohol Use Disorders Identification Test (AUDIT) total and subscale scores 23 and number of alcohol‐related harms were secondary outcomes. Mean total AUDIT score for players was reported, and the proportion of players with total AUDIT scores of 8 and above was used to categorise members as consuming alcohol at risky/high‐risk levels 23. For the AUDIT subscales, increased risk of alcohol‐related harm was defined as a hazardous use score of 6 or more (items 1–3), a dependency score of 4 or more (items 4–6) and a harmful use score of 1 or more (items 7–10) 23. Players were asked 13 questions about their experiences of alcohol‐related harm derived from a national study of the habits of Irish drinkers (Box S2) 22. The total number of harms experienced by each player was calculated, and median and mean numbers of harms for control and intervention group club players reported.

#### Player and club characteristics

Players were asked their age, highest level of education, sports that they played at the club (hurling and/or Gaelic football) and age when they had their first full alcoholic drink. Club managers were asked the number of players at the club. Clubs were classified as rural (total population of less than 1500) or urban based on geographic location.

#### Intervention fidelity and exposure

Project records were used to collect data on implementation of the intervention components and on the number of players, managers and coaches that attended intervention sessions. Club managers reported on the development and implementation of a club alcohol policy.

### Sample size and power calculations

Sample size was based on detecting a 10% reduction in the prevalence of the consumption of six or more drinks on a single occasion once a week for the repeat cross‐sectional samples. The baseline prevalence of this outcome was estimated to be 48% 22. With power of 80% and a two‐sided significance level of 5%, 760 participants (380 in the control group and 380 in the intervention group) were estimated to be required at post‐intervention. To account for clustering by club, the estimate was inflated by an intracluster correlation of 0.01 24, resulting in a required sample of 942 players at post‐intervention (38 clusters; average of 25 players per cluster).

### Statistical analysis

The primary analysis of all trial outcomes was undertaken using data from the repeat cross‐sectional samples of players at pre‐intervention and post‐intervention for those clubs that had both pre‐intervention and post‐intervention data (‘complete’ data). For all outcomes, secondary analysis was undertaken on paired data available for players who completed both pre‐intervention and post‐intervention surveys.

For all outcome measures, generalised linear mixed models were developed utilising logistic regression analyses for categorical outcomes, linear regression for continuous outcomes and negative binomial regression for count outcomes. Time and group variables were included in each model, and adjustments made for clustering at the club level through a random club‐specific intercept term. Each model controlled for age, level of education and age of onset of alcohol consumption (first full alcoholic drink) as baseline data showed these variables to be associated with alcohol use outcomes. A significance threshold of 0.05 was used for analysis of primary outcomes and 0.01 for secondary outcomes. Such analysis was undertaken for the repeat cross‐sectional samples of players and paired participant data, with an additional random subject‐specific intercept to account for repeated measurements on the same subject for the paired data.

For the cross‐sectional samples, additional sensitivity analysis was undertaken for all outcomes to assess the impact of missing data. Using multiple imputations, missing data were assigned using the chained equation method to generate a number of complete data sets 25. The imputation model included covariates considered to be associated with either the missing data or the outcome itself (level of education and age of onset of alcohol consumption) 25. Regression coefficients and standard errors from all imputed data sets were then pooled using the method reported by Rubin 26.

All statistical analyses were conducted using SAS v9.4 (SAS Institute, Cary, NC, USA).

## Results

### Participants

#### Repeat cross‐sectional samples

**Figure 1:**
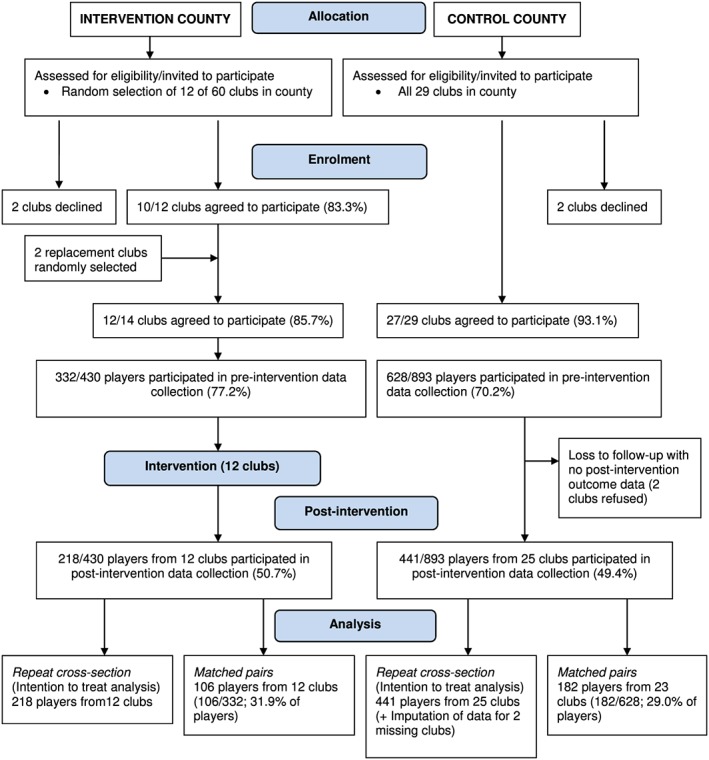
CONSORT flow chart.

Club and player participation in the trial is described in Figure 1. Of the 14 clubs from the intervention county that were randomly selected and invited to participate in the trial, 12 consented to participate (85.7%). Of the 29 clubs within the control county, 27 (93.1%) agreed to participate. Pre‐intervention data were collected from 960 players (control: *n* = 628, 70.2% consent rate; intervention: *n* = 332, 77.2% consent rate). Two control clubs were lost to follow‐up for which data were imputed. Players from 25 control clubs (*n* = 441) and all 12 intervention clubs (*n* = 218) participated in post‐intervention data collection.

#### Paired data

One‐hundred and eighty‐two players from 23 control group clubs and 106 players from 12 intervention group clubs provided both pre‐intervention and post‐intervention data.

### Sample description

Pre‐intervention, control and intervention clubs were similar in size, but differed on geographic region, with 84% of control clubs classified as rural compared with 58% of intervention clubs. Most players from both control and intervention group clubs were football players (95 and 89% respectively) with 43 and 30% respectively, playing both hurling and football. The mean age of players was 24.0 years (SD: 5.2), and the mean age at first alcoholic drink was 15.3 years (SD: 2.4). Both were similar across control and intervention groups. Refer to Table S2 for further information on clubs and players.

### Outcome analysis

#### Repeat cross‐sectional samples

Summary statistics for each primary and secondary outcome for the repeat cross‐sectional sample analysis are shown in Table 1, and the results of the generalised linear mixed models for these analyses are shown in Table 2. At pre‐intervention, around half the players reported drinking more than six drinks on at least one occasion each week (50% control group; 54% intervention group), and at post‐intervention, these proportions were similar (47% control group; 49% intervention group). At pre‐intervention, 30% of players in the control group and 31% in the intervention group reported consuming over the weekly recommendation of 21 units of alcohol per week. Post‐intervention, these proportions were 24% for control group players and 22% for intervention group players.

**Table 1:** Summary of outcome variables for cross‐sectional samples, pre‐intervention and post‐intervention

		Pre‐intervention		Post‐intervention	
Variable		Control^a^ (*n* = 591) *n* (%)	Intervention (*n* = 332) *n* (%)	Control (*n* = 441) *n* (%)	Intervention (*n* = 218) *n* (%)
Regularly >21 units alc./week	No	393 (70%)	219 (69%)	258 (76%)	112 (78%)
	Yes	170 (30%)	100 (31%)	83 (24%)	32 (22%)
Regular heavy episodic drinking	No	280 (50%)	150 (46%)	192 (53%)	109 (51%)
	Yes	285 (50%)	174 (54%)	168 (47%)	103 (49%)
Total AUDIT score (from possible 40)	Mean (SD)	12 (6)	12 (6)	11 (6)	11 (6)
	Median (range)	11 (0, 34)	11 (0, 33)	10 (0, 31)	10 (0, 36)
Total AUDIT score 8+	<8	123 (24%)	81 (27%)	104 (30%)	50 (27%)
	8 or more	390 (76%)	217 (73%)	246 (70%)	135 (73%)
AUDIT hazardous subscale score 6+	<6	30 (5.3%)	19 (5.9%)	21 (5.1%)	10 (4.8%)
	6 or more	532 (95%)	304 (94%)	388 (95%)	197 (95%)
AUDIT dependency subscale score 4+	<4	225 (40%)	125 (39%)	162 (40%)	83 (40%)
	4 or more	336 (60%)	197 (61%)	242 (60%)	124 (60%)
AUDIT harmful subscale score 1+	<1	139 (25%)	89 (28%)	124 (31%)	52 (25%)
	1 or more	426 (75%)	234 (72%)	277 (69%)	155 (75%)
Number of alcohol‐related harms	Mean (SD)	4 (3)	4 (3)	3 (3)	3 (3)
	Median (range)	4 (0, 13)	3 (0, 13)	2 (0, 13)	1 (0, 13)

AUDIT, Alcohol Use Disorders Identification Test.

a For control clubs followed up post‐intervention.

**Table 2:** Intervention effects for cross‐sectional samples at follow‐up (adjusted^a^)

		Clubs with complete data			Sensitivity analysis (imputed data)	
Outcome	ICC	OR^b^ (95% CI)	*P* value	Interaction OR^c^ (95% CI)	OR^b^ (95% CI)	*P* value
Regularly >21 units alc./week	0.02	0.81 (0.48, 1.36)	0.422	0.79 (0.44, 1.40)	0.96 (0.57, 1.60)	0.867
Regular heavy episodic drinking	0.05	1.07 (0.64, 1.79)	0.784	0.99 (0.60, 1.65)	1.09 (0.69, 1.73)	0.709
Total AUDIT^d^ score (from possible 40) (difference in means, intervention control)	0.01	0.09 (−1.19, 1.37)	0.891	0.47 (−1.02, 1.95)	0.03 (−1.13, 1.20)	0.956
Total AUDIT score 8+	0.02	1.03 (0.62, 1.70)	0.910	1.29 (0.72, 2.29)	1.11 (0.75, 1.64)	0.611
AUDIT hazardous subscale score 6+	0.03	1.26 (0.40, 3.95)	0.696	1.77 (0.49, 6.32)	0.89 (0.37, 2.12)	0.791
AUDIT dependency subscale score 4+	0.01	0.86 (0.56, 1.32)	0.501	0.86 (0.52, 1.41)	0.97 (0.67, 1.42)	0.885
AUDIT harmful subscale score 1+	0.01	1.28 (0.79, 2.07)	0.309	1.62 (0.92, 2.83)	1.26 (0.84, 1.89)	0.267
Number alcohol‐related harms	0.01	IRR 1.14 (0.93, 1.39)	0.205	IRR 1.25 (0.99, 1.57)	IRR 0.78 (0.63, 0.98)	0.029

AUDIT, Alcohol Use Disorders Identification Test; CI, confidence interval; ICC, intraclass correlation; IRR, incident rate ratio; OR, odds ratio.

a Adjusted for age, age of onset of alcohol use and Leaving Certificate.

b Odds ratio: intervention versus control post‐intervention.

c Odds ratio: difference in how the outcomes changed over time.

The proportion of players that reported a total AUDIT score above 8 was similar across both groups pre‐intervention (control: 76%; intervention: 73%) and post‐intervention (control: 70%; intervention: 73%). Based on the AUDIT subscales, at pre‐intervention, almost all players (control: 95%; intervention: 94%) were assessed as drinking at hazardous levels, three‐quarters at levels that placed them in harmful situations (control: 75%; intervention: 72%) and 60% at risk of alcohol dependence (control: 60%; intervention: 61%). For all AUDIT subscale measures, post‐intervention proportions remained similar to pre‐intervention proportions.

There were no significant between‐group differences at follow‐up for any of the primary or secondary outcome variables for those players whose clubs had complete repeat cross‐sectional data. Results from the sensitivity analysis of the imputed data sets similarly showed no significant between‐group differences for most outcomes, apart from number of alcohol‐related harms (Table 2). This analysis found a statistically significant lower median number of alcohol‐related harms amongst intervention group players (median: 1; range 0–13) compared with control group players (median: 2; range 0–13) at post‐intervention [IRR 0.78 (0.63, 0.98); *P* = 0.029] (Table 2).

#### Paired data

There were 288 individual players with data available at pre‐intervention and post‐intervention. Summary statistics for these players are reported in Table 3 and results of the generalised linear mixed models in Table 4. There were no statistically significant differences in AUDIT scores or consumption of six drinks on at least one occasion each week or >21 units of alcohol per week, although both were lower in the intervention group at follow‐up.

**Table 3:** Summary of outcome variables for paired data pre‐intervention and post‐intervention

		Pre‐intervention		Post‐intervention	
Variable		Control (*n* = 182)	Intervention (*n* = 106)	Control (*n* = 182)	Intervention (*n* = 106)
Regularly >21 units alc./week	No	116 (67%)	76 (74%)	110 (71%)	56 (79%)
	Yes	56 (33%)	27 (26%)	45 (29%)	15 (21%)
Regular heavy episodic drinking	No	91 (52%)	50 (49%)	80 (49%)	57 (55%)
	Yes	84 (48%)	52 (51%)	83 (51%)	47 (45%)
Total AUDIT score (from possible 40)	Mean	12 (6)	12 (7)	11 (6)	11 (5)
	Median	11 (0, 34)	11 (0, 33)	11 (0, 31)	10 (0, 29)
Total AUDIT score 8+	<8	36 (22%)	30 (31%)	47 (30%)	26 (29%)
	8 or more	127 (78%)	66 (69%)	111 (70%)	65 (71%)
AUDIT hazardous subscale score 6+	<6	6 (3.5%)	8 (7.7%)	3 (1.8%)	7 (6.9%)
	6 or more	166 (97%)	96 (92%)	167 (98%)	95 (93%)
AUDIT dependency subscale score 4+	<4	63 (37%)	48 (46%)	61 (36%)	44 (43%)
	4 or more	109 (63%)	56 (54%)	108 (64%)	58 (57%)
AUDIT harmful subscale score 1+	<1	41 (24%)	34 (33%)	44 (26%)	34 (33%)
	1 or more	132 (76%)	70 (67%)	125 (74%)	68 (67%)
Number of reported alcohol‐related harms	Mean	4 (3)	3 (3)	3 (3)	2 (3)
	Median	4 (0, 13)	3 (0, 13)	3 (0, 13)	0 (0, 11)

AUDIT, Alcohol Use Disorders Identification Test.

**Table 4:** Intervention effects for paired data at follow‐up

Variable	Interaction OR^a^ (95% CI)	OR^b^ (95% CI)	*P* value
Regularly >21 units alc./week	0.92 (0.34, 2.45)	0.62 (0.27, 1.41)	0.250
Regularly heavy episodic drink	0.64 (0.28, 1.49)	0.75 (0.35, 1.59)	0.447
Total AUDIT score (difference in means, intervention control)	−0.06 (−1.46, 1.34)	−0.55 (−2.04, 0.93)	0.454
AUDIT score >8	3.24 (0.93, 11.27)	1.18 (0.31, 4.46)	0.802
AUDIT score hazardous score 6+	0.54 (0.00, 693.40)	0.25 (0.00, 236.10)	0.688
AUDIT score dependency score 4+	1.11 (0.47, 2.64)	0.61 (0.27, 1.40)	0.244
AUDIT score harmful score 1+	1.17 (0.41, 3.39)	0.50 (0.16, 1.52)	0.220
Number of alcohol‐related harms	IRR 0.70 (0.51, 0.94)	IRR 0.56 (0.37, 0.84)	0.005

AUDIT, Alcohol Use Disorders Identification Test; CI, confidence interval; IRR, incident rate ratio; OR, odds ratio.

a Odds ratio: intervention versus control post‐intervention

b Odds ratio: difference in how the outcomes changed over time.

There was a statistically significant reduction in the number of alcohol‐related harms reported by players in the intervention group at post‐intervention compared with players in the control group (IRR = 0.56, *P* = 0.005) (Table 4).

### Intervention fidelity and exposure

Player and manager/coach participation in the various components of the intervention was low. Just over half of the players (*n* = 115, 52.7%) from intervention group clubs that were surveyed post‐intervention reported that they attended the alcohol training session, and 14.2% (*n* = 31) reported being aware of the awareness campaign.

Two‐thirds (*n* = 8) of the 12 intervention club managers attended the alcohol training session, and half (*n* = 6) attended the alcohol policy session. None of the clubs had a written alcohol policy prior to the intervention, and one‐third (*n* = 4) had one in place after the intervention. All 12 of the managers were aware of the intervention being conducted at their club. Thirteen coaches from a quarter of the clubs (*n* = 3) attended the alcohol education session for coaches.

## Discussion

This is only the second study globally to report the outcomes of an alcohol harm reduction intervention in community sports clubs. Post‐intervention, there was no significant difference between intervention and control group club players in the proportion consuming six or more drinks on at least one occasion per week, drinking more than the weekly recommended limit of 21 units of alcohol per week or with total AUDIT or AUDIT subscale scores indicative of risk of alcohol‐related harm. There was, however, for players with both pre‐intervention and post‐intervention (paired) data, a statistically significant difference, post‐intervention, in the median number of alcohol‐related harms reported by players of intervention clubs compared with players of control group clubs [IRR 0.56 (0.37, 0.84); *P* = 0.005].

The findings of this trial differ, in part, from those of the trial conducted by Kingsland *et al*. with community football clubs in Australia. Compared with the findings of this trial, the intervention reported by Kingsland *et al*. was found to be effective in reducing risky alcohol consumption by community football club members at the club as well as risk of alcohol‐related harm overall 19. The contrasting results may be the result of a number of differences between the studies. First, the participants differed across the two trials. All participants in the current trial were players, whereas the participants in the Kingsland *et al*. trial were a mixture of players (53%), fans/supporters (16%) and club management/coaches/other (30%). While the Kingsland *et al*. trial did not report effectiveness for these participant groups individually, there is the potential that alcohol management interventions implemented at the club bar may have been more effective for groups other than players 27.

Second, the implementation of the intervention in the Kingsland *et al*. trial was supported by a suite of practice change support strategies that were not included in the current study, such as observational audit and feedback, accreditation, cost recovery and support from sporting associations 28. Implementation science theory asserts that such strategies are important for interventions to be effectively implemented and achieve maximum participant uptake 29.

Third, the intervention in the trial conducted by Kingsland *et al*. was implemented over 2.5 years compared with the 4 month period in this study. The longer period of the Kingsland *et al*. intervention may have resulted in greater club member exposure to the intervention and hence greater impact on levels of alcohol consumption and related harms. Both time and dose have been found to be associated with intervention effectiveness and may account for some of the differential effect between the two studies 30.

Low levels of intervention participation and acceptability may have also contributed to the ineffectiveness of the intervention. Only 52.7% of the players in the intervention group attended the alcohol training session, and only 14.2% reported knowledge of the awareness campaign. Attendance at the coach training session was also low with only 13 coaches (from three clubs) attending the training session. As the coaches found the alcohol training session useful, higher uptake amongst the clubs may have had more impact on alcohol outcome measures. Furthermore, although a majority of the managers (83.3%) reported that they found the intervention useful, only 4 of the 12 clubs had implemented a written alcohol policy at the end of the intervention, an important environmental strategy to support the other education‐based intervention strategies. Future alcohol harm reduction trials in the sports setting should seek to increase the implementation of socio‐environmental strategies, which theory and evidence suggest are important in modifying alcohol use behaviour 17. Such trials should also test the effectiveness of individual strategies so that interventions can continue to be refined for maximum effectiveness and efficiency.

Despite these factors, the positive intervention effect for the alcohol‐related harm outcome suggests the intervention was of sufficient intensity and duration to impact on this measure. Possible explanations for the differential findings by outcome measure may be the greater statistical power obtained from a repeated measures analytical approach for the paired count data or the greater appeal of the intervention content focussing on the avoidance of harms rather than on alcohol consumption *per se*. This finding, together with unpublished evidence of GAA clubs independently implementing harm reduction measures and limiting alcohol industry sponsorship, suggests that re‐designed alcohol harm reduction program trials are warranted and feasible.

While the controlled study design and the randomisation of intervention county clubs were strengths of the study, a number of limitations need to be noted. Counties were not randomly assigned to control and intervention groups, and this may have resulted in the outcomes being confounded by factors that were not controlled for in the analysis. The study only included male players, and as such, the findings cannot be generalised to female players, and the study was not powered to assess any differential intervention effects by level of player professionalism or rural/urban location. Differences in the length of pre‐intervention (May 2006 to April 2008) and post‐intervention (May to October 2008) data collection periods should also be noted. While it is unknown how these differences may have affected the trial outcomes, periods of similar length and seasonality might have elicited a different result. Finally, the study may have been underpowered to detect effects of the intervention given that the final participant numbers were substantially lower than the predicted required sample size.

Given the limited number of controlled trials of interventions to reduce alcohol misuse and alcohol‐related harm in the sports setting, and the different findings of the two that have been conducted, further high‐quality trials are required to determine if such interventions are feasible and effective. These trials should include greater focus on strategies to support implementation, such as those employed by Kingsland *et al*., a longer implementation period and strategies focused on modifying the drinking environment as well as providing training to players and clubs.

## Conflict of interest

None to declare.

## Supporting information


**Table S1:** Intervention content and format

**Table S2:** Comparison of club and player characteristics at baseline

**Box S1:** Measurement of primary outcomes

**Box S2:** Measurement of player experience of alcohol‐related harms

## References

[1] Department of Health and Children. Strategic Taskforce on Alcohol . Second Report. Dublin, Ireland: Department of Health and Children; 2004.

[2] Organisation for Economic Co‐operation and Development . OECD Health Statistics 2014 [09 May 2016]. Available from: http://www.oecd.org/els/health‐systems/oecd‐health‐statistics‐2014‐frequently‐requested‐data.htm.

[3] Morgan K , McGee H , Watson D , Perry I , Barry M , Shelley E . SLAN 2007: survey of lifestyle, attitudes and nutrition in Ireland. Main Report Dublin, Ireland: Department of Health and Children, 2008.

[4] Department of Health . Healthy Ireland Survey 2015: summary of findings. Dublin: 2015.

[5] Wechsler H . Binge drinking, tobacco, and illicit drug use and involvement in college athletics. J Am Coll Health 1997;45:195–200. 9069676 10.1080/07448481.1997.9936884

[6] O'Brien KS , Blackie JM , Hunter JA . Hazardous drinking in elite New Zealand sportspeople. Alcohol Alcohol 2005;40:239–241. 15797880 10.1093/alcalc/agh145

[7] Black D , Lawson J , Fleishman S . Excessive alcohol use by non‐elite sportsmen. Drug Alcohol Rev 1999;18:201–205.

[8] Wechsler H , Davenport AE . Binge drinking, tobacco, and illicit drug use and. J Am Coll Health 1997;45:195. 9069676 10.1080/07448481.1997.9936884

[9] Bedendo A , Opaleye ES , Andrade ALM , Noto AR . Heavy episodic drinking and soccer practice among high school students in Brazil: the contextual aspects of this relationship. BMC Public Health 2013;13:247. 23514566 10.1186/1471-2458-13-247 PMC3610150

[10] Brenner J , Swanik K . High‐risk drinking characteristics in collegiate athletes. J Am Coll Health 2007;56:267–272. 18089508 10.3200/JACH.56.3.267-272

[11] Martha C , Grelot L , Peretti‐Watel P . Participants' sports characteristics related to heavy episodic drinking among French students. Int J Drug Policy 2009;20:152–160. 18201884 10.1016/j.drugpo.2007.11.023

[12] Sønderlund AL , O'Brien K , Kremer P , *et al.* The association between sports participation, alcohol use and aggression and violence: a systematic review. J Sci Med Sport 2014;17:2–7. 23602563 10.1016/j.jsams.2013.03.011

[13] O'Farrell A , Allwright S , Kenny S , Roddy G , Eldin N . Alcohol use among amateur sportsmen in Ireland. BMC Res Notes 2010;3:313. 21087506 10.1186/1756-0500-3-313 PMC3000422

[14] Holder HD , Saltz R , Grube J , *et al.* Summing up: lessons from a comprehensive community prevention trial. Addiction 1997;92:S293–S301. 9231452

[15] Holder HD . A community systems approach to alcohol problem prevention. Cambridge, England: Cambridge University Press, 1997.

[16] Wagenaar AC , Murray DM , Toomey TL . Communities mobilizing for change on alcohol (CMCA): effects of a randomized trial on arrests and traffic crashes. Addiction 2000;95:209–217. 10723849 10.1046/j.1360-0443.2000.9522097.x

[17] Babor T , Caetano R , Casswell S , *et al.* Alcohol: no ordinary commodity—research and public policy. Oxford: Oxford University Press, 2010.

[18] Priest N , Armstrong R , Doyle J , Waters E . Policy interventions implemented through sporting organisations for promotiong healthy behaviour change. Cochrane Database Syst Rev 2008;3(Art. No.pub:CD004809 https://doi.org/10.1002/14651858. CD004809. 10.1002/14651858.CD004809.pub3 PMC6464902 18646111

[19] Kingsland M , Wolfenden L , Tindall J , *et al.* Tackling risky alcohol consumption in sport: a cluster randomised controlled trial of an alcohol management intervention with community football clubs. J Epidemiol Community Health 2015;69:993–999. 26038252 10.1136/jech-2014-204984 PMC4602266

[20] Gaelic Athletics Association . About the GAA[August 2015]. Available from: http://www.gaa.ie/the‐gaa/about‐the‐gaa/.

[21] Cahalan D , Cisin I . American drinking practices: summary of findings from a national probability sample. II. Measurement of massed versus spaced drinking. Q J Stud Alcohol 1968;29:642–656. 5682663

[22] Ramstedt M , Hope A . The Irish drinking habits of 2002. Drinking and drinking related harm, a European perspective. J Subst Use 2005;10:271–283.

[23] Saunders J , Aasland O , Babor T , De La Fuente J , Grant M . Development of the Alcohol Use Disorders Identification Test (AUDIT): WHO collaborative project on early detection of persons with harmful alcohol consumption. Addiction 1993;88:791–804. 8329970 10.1111/j.1360-0443.1993.tb02093.x

[24] Campbell M , Donner A , Klar N . Developments in cluster randomized trials and Statistics in Medicine. Stat Med 2007;26:2–19. 17136746 10.1002/sim.2731

[25] White I , Horton N , Carpenter J , Pocock S . Strategy for intention to treat analysis in randomised trials with missing outcome data. BMJ 2011;342:d40. 21300711 10.1136/bmj.d40 PMC3230114

[26] Rubin DB . Multiple imputation for nonresponse in surveys. Hoboken, New Jersey: John Wiley & Sons, 2004.

[27] Dietze PM , Fitzgerland JL , Jenkinson RA . Drinking by professional Australian Football League (AFL) players: prevalence and correlates of risk. Med J Australia 2008;189:479–483. 18976186 10.5694/j.1326-5377.2008.tb02138.x

[28] Kingsland M , Wolfenden L , Tindall J , *et al.* Improving the implementation of responsible alcohol management practices by community sporting clubs: a randomised controlled trial. Drug Alcohol Rev 2015;34:447–457. 25735650 10.1111/dar.12252

[29] Damschdoder LJ , Aron DC , Keith RE , Kirsh SR , Alexander JA , Lowery JC . Fostering implementation of health services research findings into practice: a consolidated framework for advancing implementation science. Implement Sci 2009;4:50 https://doi.org/10.1186/1748‐5908‐4‐50. 19664226 10.1186/1748-5908-4-50 PMC2736161

[30] Durlak J , DuPre E . Implementation matters: a review of research on the influence of implementation on program outcomes and the factors affecting implementation. Am J Community Psychol 2008;41:327–350. 18322790 10.1007/s10464-008-9165-0

