# Supplementary material for: A multi‐faceted intervention to reduce alcohol misuse and harm amongst sports people in Ireland: A controlled trial
Source: Drug Alcohol Rev. 2017 Aug 7;37(1):14–22. doi: 10.1111/dar.12585 (PMC5811829; doi:10.1111/dar.12585)
Supplement: Supplementary file 1 — Table S1: Intervention content and format [file DAR-37-14-s001.docx]

**Table S1: Intervention content and format**

| **Intervention component** | **Content** | **Format** |
| --- | --- | --- |
| 1. Alcohol education for players | - Alcohol use recommendations - Harmful drinking - Drinking and sport - Harm reduction | - 1 × 10 minute introduction presentation - 1 × 60 minute alcohol education presentation + discussion (10 min introduction + 40 min presentation and 10 min question and answer session) |
| 1. Alcohol education for coaches/managers | - Identifying alcohol-related problems - Tackling alcohol-related problems among players, including addressing alcohol use culture within club - Responsible service of alcohol at club bars | - 1 × 40 minute presentation + discussion and 20 min question and answer session - Hand-outs |
| 1. Alcohol policy workshop for club managers | - Developing a written alcohol management policy for a Gaelic Athletic Association club - Current liquor licensing laws | - 1 × 10 minute presentation - 1 × 40 minute workshop on writing an alcohol policy |
| 1. Awareness campaign: “Less Pints, More Points” | - Impact of alcohol use on health and sports performance - Details of intervention | - Advertisements/posters in club dressing rooms and bars, on match programs and club websites |
