# Supplementary material for: A multi‐faceted intervention to reduce alcohol misuse and harm amongst sports people in Ireland: A controlled trial
Source: Drug Alcohol Rev. 2017 Aug 7;37(1):14–22. doi: 10.1111/dar.12585 (PMC5811829; doi:10.1111/dar.12585)
Supplement: Supplementary file 2 — Table S2: Comparison of club and player characteristics at baseline [file DAR-37-14-s004.docx]

**Table S2: Comparison of club and player characteristics at baseline**

| **Characteristic** |  | **Control^a^** | **Intervention** | **Total** |  |
| --- | --- | --- | --- | --- | --- |
| **Clubs** |  | **n=25** | **n=12** | **N=39** |  |
| Large club  (>40 Players) | No | 19 (76%) | 8 (67%) | 27 (73%) |  |
|  | Yes | 6 (24%) | 4 (33%) | 10 (27%) |  |
| Number of registered players | Mean (SD) | 34 (11) | 36 (11) | 34 (11) |  |
|  | Median (min, max) | 30 (25, 60) | 31 (25, 60) | 30 (25, 60) |  |
| Geographic region | Rural | 21 (84%) | 7 (58%) | 28 (76%) |  |
|  | Urban | 4 (16%) | 5 (42%) | 9 (24%) |  |
| **Players** |  | **n=591** | **n=332** | **N=923** |  |
| Attained Leaving Certificate | No | 150 (26%) | 62 (19%) | 212 (23%) | |
|  | Yes | 429 (74%) | 268 (81%) | 697 (77%) | |
| Hurling player | No | 390 (66%) | 154 (46%) | 544 (59%) | |
|  | Yes | 201 (34%) | 178 (54%) | 379 (41%) | |
| Football player | No | 30 (5.1%) | 37 (11%) | 67 (7.3%) | |
|  | Yes | 561 (95%) | 295 (89%) | 856 (93%) | |
| Hurling and football player | No | 414 (70%) | 190 (57%) | 604 (65%) | |
|  | Yes | 177 (30%) | 142 (43%) | 319 (35%) | |
| Age of player | Mean (SD) | 23.8 (5.2) | 24.3 (5.2) | 24.0 (5.2) | |
|  | Median (min, max) | 22.8 (16.0, 46.5) | 23.9 (16.0, 41.4) | 23.0 (16.0, 46.5) | |
| Age at first alcoholic drink | Mean (SD) | 15.4 (2.5) | 15.1 (2.1) | 15.3 (2.4) | |
|  | Median (min, max) | 15.5 (5.0, 42.0) | 15.0 (5.0, 30.0) | 15.0 (5.0, 42.0) | |

**^a^** Baseline characteristics for the 25 control clubs that provided data at post-intervention.
