# Supplementary material for: A multi‐faceted intervention to reduce alcohol misuse and harm amongst sports people in Ireland: A controlled trial
Source: Drug Alcohol Rev. 2017 Aug 7;37(1):14–22. doi: 10.1111/dar.12585 (PMC5811829; doi:10.1111/dar.12585)
Supplement: Supplementary file 3 — Box S1: Measurement of primary outcomes [file DAR-37-14-s003.docx]

**Box S1: Measurement of primary outcomes**

To measure frequency of consumption for each of four beverage types (beer/cider/wine/spirits), participants were asked: “During the past 12 months how often did you usually drink beer/cider/wine/spirits?”. The response options were: “Every day”, “4-5 times per week”, “2-3 times per week”, “Once a week”, “2-3 times per month”, “Once a month”, “Less often than once a month”, “Never”. To measure quantity of consumption, participants were asked, for each beverage type: “When you drink beer/cider/wine/spirits, how much do you usually drink?” The responses for beers/ciders were “Half pints”, “Pints”, “Small cans” and “Large cans”; for wine were “Glasses”, “Quarter bottles” and “Bottles”; and for spirits were “Single measure of spirit”, “Single shot” and “Bottle of pre-mixed spirits”.

The proportion of players who reported drinking six or more standard alcoholic drinks in one sitting at least once a week was determined by asking: “During the last month, how many times have you had six or more standard drinks in a row?” A standard drink was defined as one glass beer/lager/cider, a glass of wine, a measure of spirits. A pint of beer/ lager or stout was defined as two drinks. The response options were: “Never”, “Once a month”, “Twice a month”, “3-5 times per month”, “6 to nine times per month” or “10 or more times per month”.
