# Supplementary material for: A multi‐faceted intervention to reduce alcohol misuse and harm amongst sports people in Ireland: A controlled trial
Source: Drug Alcohol Rev. 2017 Aug 7;37(1):14–22. doi: 10.1111/dar.12585 (PMC5811829; doi:10.1111/dar.12585)
Supplement: Supplementary file 4 — Box S2: Measurement of player experience of alcohol‐related harms [file DAR-37-14-s002.docx]

**Box S2: Measurement of player experience of alcohol-related harm**

Players were asked whether they had experienced any of the following in the last 12 months:

1. A fight due to their drinking
2. An accident due to their drinking
3. Attended accident and emergency due to their drinking
4. Missed time from work/college due to their drinking
5. Felt they should cut down on their drinking
6. Regretted something said or done due to their drinking
7. Felt that drinking harmed their home life/marriage/ relationship
8. Felt that drinking harmed their work/studies
9. Felt that drinking harmed their friendships/social life
10. Felt that drinking harmed their health
11. Felt that they were verbally abusive due to their drinking
12. Damaged public property because of their drinking
13. Were physically sick because of their drinking
